# Relation between hand function and gross motor function in full term infants aged 4 to 8 months

**DOI:** 10.1590/bjpt-rbf.2014.0070

**Published:** 2015

**Authors:** Solange F. Nogueira, Elyonara M. Figueiredo, Rejane V. Gonçalves, Marisa C. Mancini

**Affiliations:** 1 Terapeuta Ocupacional, Belo Horizonte, MG, Brazil; 2 Departamento de Fisioterapia, Universidade Federal de Minas Gerais (UFMG), Belo Horizonte, MG, Brazil; 3 Programa de Pós-graduação em Ciências da Reabilitação, UFMG, Belo Horizonte, MG, Brazil; 4 Departamento de Terapia Ocupacional, UFMG, Belo Horizonte, MG, Brazil

**Keywords:** child development, motor skills, motor activity, rehabilitation

## Abstract

**Background:**

In children, reaching emerges around four months of age, which is followed by rapid changes in hand function and concomitant changes in gross motor function, including the acquisition of independent sitting. Although there is a close functional relationship between these domains, to date they have been investigated separately.

**Objective:**

To investigate the longitudinal profile of changes and the relationship between the development of hand function (i.e. reaching for and manipulating an object) and gross motor function in 13 normally developing children born at term who were evaluated every 15 days from 4 to 8 months of age.

**Method:**

The number of reaches and the period (i.e. time) of manipulation to an object were extracted from video synchronized with the Qualisys^(r)^ movement analysis system. Gross motor function was measured using the Alberta Infant Motor Scale. ANOVA for repeated measures was used to test the effect of age on the number of reaches, the time of manipulation and gross motor function. Hierarchical regression models were used to test the associations of reaching and manipulation with gross motor function.

**Results:**

Results revealed a significant increase in the number of reaches (p<0.001), the time of manipulation (p<0.001) and gross motor function (p<0.001) over time, as well as associations between reaching and gross motor function (R^2^=0.84; p<0.001) and manipulation and gross motor function (R^2^=0.13; p=0.02) from 4 to 6 months of age. Associations from 6 to 8 months of age were not significant.

**Conclusion:**

The relationship between hand function and gross motor function was not constant, and the age span from 4 to 6 months was a critical period of interdependency of hand function and gross motor function development.

## Introduction

Interest in the relationship between global and intentional movement of the upper limbs in children may be due to the close functional relationship observed between these two subsystems^1^^,^ 2. Contemporary approaches suggest that motor actions in the child are derived from the interaction between multiple subsystems, and these actions organize themselves in real time to meet the task demands and contextual conditions^3^^,^ 4. From this perspective, changes in child development result from the interplay of multiple factors, which may alternate in different developmental periods and be combined in unprescribed ways^5^^,^ 6.

In infants, the development of hand and gross motor functions have been investigated separately^7^^-^ 10 despite the concomitant development and intrinsic interrelationship of these acquisitions^11^^,^ 12. It is believed that in the first year of life, gross motor acquisitions are the basis for hand function improvements, such as reaching and manipulation of an object^13^. This argument suggests the existence of a moderate or strong positive association between gross motor function and hand function. However, some authors have reported weak correlations^14^^,^ 15. Darrah et al.^16^ assessed gross and fine motor development among 120 Canadian children at the ages of 9, 11, 13, 16 and 21 months, and found a weak positive association between these two motor domains at the end of the first and during the second year of life. The authors argued that gross and fine motor skills follow different developmental trajectories and have different emergence rates^16^. Based on this scenario, gross and fine motor skills would be expected to develop simultaneously and show a moderate association between them^16^.

Hand and gross motor functions are important because these domains are included in the assessment of child development. Assumptions about the relationship between these domains are not based on empirical research but rather on inferences regarding their effects on one another. Thus, it is necessary to assess this relationship and provide evidence to support or reject the arguments reported in the literature.

The present study aimed to assess the longitudinal profile of changes and the association between gross motor and hand function (i.e. reaching and manipulation of an object) development in children age 4 to 8 months who were born at term. Its novelty relies on the investigation of the correlation between hand and gross motor functions, starting at the emergence of hand function (at 4 months)^7^ and continuing over the period in which rapid changes occur both in this domain and in gross motor function, including acquisition of independent sitting. Therefore, the intervals between repeated measurements were short (15 days) and aimed at identifying the profile of changes in hand function development and the relationship between hand function development and gross motor function development. Our hypothesis was that a mild or moderate association existed between hand and gross motor function developments among infants age 4 to 8 months.

## Method

### Participants

The sample size was calculated based on the results of a longitudinal study that investigated the development of reaching among children 19 to 31 weeks of age, which demonstrated an effect size of d=1.86 for the number of reaches^7^. To replicate this effect, a sample size of 10 to 12 children was estimated. Thirteen children born at term, with appropriate weight for gestational age (6 boys and 7 girls; mean weight at birth=3,447 g, SD=414 g; mean gestational age of 39 weeks, SD=0.8 weeks) were non-randomly recruited via the authors' personal contact with pediatricians and other individuals from the academic community, and these children were assessed biweekly from 4 to 8 months of age. Children with neonatal complications were excluded. The Universidade Federal de Minas Gerais (UFMG) Ethics Committee, Belo Horizonte city, state of Minas Gerais-MG, Brazil (ETIC 326/05) approved the study, and the parents provided informed consent.

### Procedures and instrumentation

**Figure 1:**
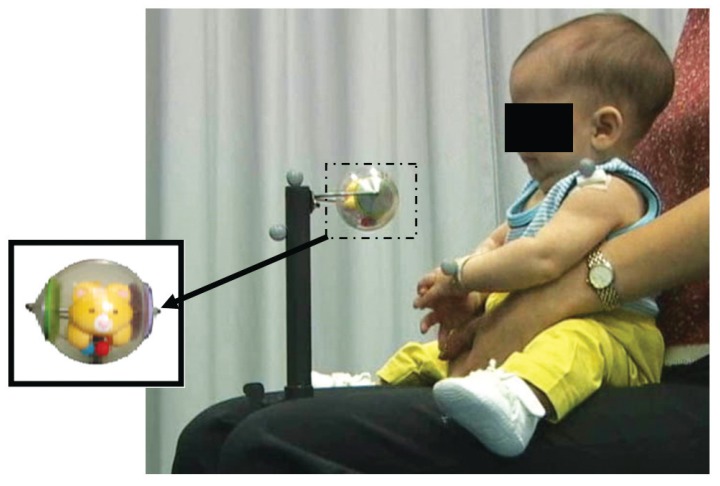
Infant positioned during the manual function evaluation with the object expanded.

In each biweekly assessment, kinematic data of reaching and manipulation of an object were collected to document hand function (i.e. number of reaches and amount of time of manipulating/touching the object). The Qualisys Pro-Reflex^(r)^ motion capture system was used with two cameras positioned on each side of the child, and upper limb movements were recorded at a frequency of 120 Hz. Reflective markers with 1.5-cm diameters were placed on each shoulder (1 cm below the acromion) with double-sided adhesive tape and on each wrist (aligned with the third metacarpal bone) with elastic straps. In addition, two digital video cameras (8 mm) were positioned on the right and left diagonals to record the child's reaching behavior at a frequency of 30 Hz. The object was a transparent sphere with a 5.8-cm diameter that contained one dog and three colored balls (Fisher-Price^(r)^ brand), fixed on the highest point of an adjustable-height metal rod that was placed on the floor. When touched, the object spun around the metal rod's axis, in the frontal plane, with subsequent movement of the three balls and sound emission (Figure 1). The environmental conditions were adjusted to facilitate the child's performance (i.e., minimum noise level, ambient light and mild temperature).

The biweekly assessments of the children's gross motor function were performed with the Alberta Infant Motor Scale (AIMS). AIMS is a predominantly observational standardized scale that records child gross motor performance from birth through 18 months of age^17^. It comprises 58 items that record the child's spontaneous movement repertoire in the following four positions: prone (21 items), supine (9 items), sitting (12 items) and standing (16 items). The total raw score and the child's age are entered into a chart, which reports the gross motor development percentile. This scale has been used in both national and international studies and has high levels of reliability and validity^18^^-^ 20.

A caregiver sat in a chair in front of the object and held the child on his or her lap^21^. The object was adjusted to align with the child's shoulder. The caregiver was instructed to hold the child by the hips without interfering with upper limb movements. The protocol used to assess upper limb movement was described by Berthier and Keen^21^. The object was initially concealed from the child and uncovered immediately prior to data collection. The motion capture system, which was synchronized with two digital video cameras, was turned on when the child directed his or her eyes and attention to the object. During the assessments, it was assured that the child was alert and not crying, and if the child did not attempt to reach the object, the examiner spun it to draw the child's attention. Data collection lasted 1.5 minutes, and gross motor function was assessed with the AIMS scale. One examiner who was experienced in this scale and demonstrated intra-examiner agreement of 0.95 administered the AIMS scale to all participants.

### Data reduction

The number of times that the child reached the object and the time spent manipulating the object during the data collection period were obtained by video analysis using the BSplayer Pro File 2.4 software. The start of reaching was defined as the first visually detectable movement of the hand towards the object that resulted in touch^22^. The end of reaching was identified by the first frame in which the hand or any finger touched the object^4^. The start of the manipulation of the object was defined in the same way as the end of reaching. Manipulation time included the time in which the child remained in contact with the object and explored it without clearly moving the hand away. The end of manipulation of the object was defined as the first frame from which the child explicitly and continuously moved the hand away from the object.

Considering the large amount of data involved in this study, five examiners participated in data reduction. These examiners were previously trained, and data reduction began after adequate agreement levels were reached (Kappa >0.8 for identifying reaching for and manipulation of the object, and ICC>0.8 for identifying the frames corresponding to the start and end of each reach and time of manipulation).

The number of reaches for each child was calculated as the sum of reaching movements performed by the right and left upper limbs during the 1.5-minute period of longitudinal data collection. The time of manipulation was expressed in milliseconds and was calculated using Matlab^(r)^ by summing the times of manipulation of each upper limb in the same period.

The total raw score of the gross motor function assessment and the chronological age of the child in each longitudinal assessment were converted to a percentile score.

### Data analysis

Repeated measures ANOVA was used to assess the effect of age on the number of reaches, the time of manipulation of the object and gross motor function. Post-hoc analysis (pre-planned contrasts) showed bivariate differences between two ages. The level of significance was corrected for the number of post-hoc comparisons (n=10) and set at α=0.005.

Hierarchical linear regression models for longitudinal data were used to assess the association between hand function (number of reaches and time of manipulation of the object) and gross motor function. These models analyzed the variable at two levels. The first level considered the relationship between the response variable and the explanatory variable to analyze the variation between individuals. The second level considered the individual's association structure established in the first level over time, which added information on intra-individual variation.

Three models were used to assess the correlation between gross motor function (predictor variable) and the number of reaches (response variable) in the 4 to 6 months and 6 to 8 months periods, and over the nine assessments. Another three models were used to assess the association between time of manipulation (response variable) and gross motor function (explanatory variable) in the same period. The adjusted xtmixed command in STATA^(r)^ version 9.1 and the SPSS^(r)^ version 12.0 package were used for data analysis. A level of significance of α=0.05 was used in the hierarchical models.

## Results

The participants completed nine assessments in this longitudinal study. Only one 5-month-old child did not attend one of the assessments. In order for this child to be retained in the analysis, the age with missing data was considered null for the longitudinal analysis. Table 1 shows the mean values, standard deviation, minimum and maximum values for the variables number of reaches, time of manipulation of the object and gross motor function (AIMS percentile) in all ages assessed.

**Table 1:** Mean, standard deviation (SD), minimum and maximum values from the gross motor function scores, number of reaches and time of manipulation of an object on nine longitudinal evaluations.

Age (Months) Variables	4	4.5	5	5.5	6	6.5	7	7.5	8
Gross Motor Function* (AIMS Percentile)	43 (19) (8-75)	43 (17) (10-65)	45 (17) (15-78)	41 (20) (16-78)	55 (25) (16-91)	54 (23) (18-93)	57 (19) (16-94)	56 (20) (20-93)	56 (24) (21-93)
Number of Reaches*	13 (11) (0-32)	28 (17) (4-64)	34 (18) (0-69)	41 (17) (13-73)	45 (16) (20-76)	33 (13) (10-56)	49 (19) (28-85)	52 (21) (20-81)	40 (26) (7-99)
Time of Manipulation (ms)*	21 (21) (0-55)	35 (27) (0-71)	49 (24) (0-74)	57 (15) (33-82)	50 (12) (27-64)	50 (23) (0-86)	48 (15) (25-78)	46 (16) (20-67)	50 (20) (21-82)

* The first line refers to mean and (standard deviation) values with minimum and maximum values underneath, for each variable.

### Age effect on hand and gross motor functions

There was a significant increase in the number of reaches (p<0.001), the time of manipulation of the object (p<0.001) and gross motor function (p<0.001) over the longitudinal follow-up period of this study.

Post-hoc comparisons showed significant differences (p<0.005) in the number of reaches among children at 4 months of age compared with the number of reaches at the other assessments (5, 6, 7 and 8 months), as well as the number of reaches when comparing the 5^th^ month with the 7^th^ month (p=0.003).

**Figure 2:**
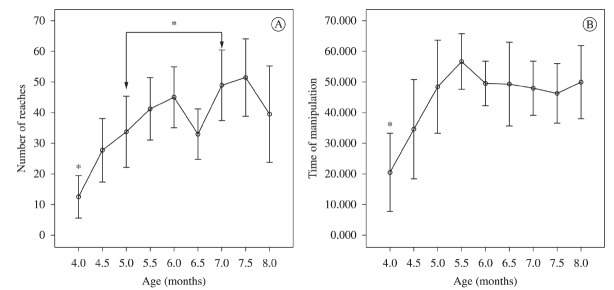
Mean values and error bars of (A) number of reaches (B) time of manipulation (ms) of an object from infants on the nine longitudinal evaluations. *Effects where p<0.005; in (A) significant differences in the comparisons of 4 months with 5, 6, 7 and 8 months; in (B) significant difference in the comparisons of 4 months with 6, 7 and 8 months.

Figure 2 shows the mean values and standard deviation of the two hand function variables for each age. The visual analysis of the mean number of reaches (Figure 2A) shows two distinct moments of this variable in the period between 4 and 8 months, with a progressive increase from 4 to 6 months, a decrease at 6.5 months, a subsequent increase at 7 and 7.5 months and a decrease at 8 months of age.

Post-hoc comparisons showed significant differences in the time of manipulation of children at 4 months compared with 6, 7 and 8 months (p=0.003). Figure 2B presents the increase in mean time of manipulation of the object between the initial age and the remaining ones. Visual analysis of this chart suggests a period of continuous increase in mean time of manipulation of the object up to 5.5 months of age, with a mild reduction of this time at 6 months; following 6 months it remains constant.

Post-hoc comparisons also revealed a difference between gross motor function at 5 and 7 months of age (p=0.002).

### Relationship between hand function and gross motor function

The follow-up period between 4 and 8 months seems to consist of two sub-phases, which are split around the 6^th^ month. To better understand the profile of changes, the relationship between hand function and gross motor function was assessed from 4 to 8 months, as well as in each sub-phase (4 to 6 and 6 to 8 months).

Three regression models were used to assess the correlation between number of reaches and gross motor function. The first model showed strong correlation for the 4 to 6 months period (p<0.001). This model explained 84.94% of the variability in the number of reaches, with 38.8% of the total variability being attributed to the inclusion of the variable gross motor function in the model. The model that assessed the correlation in the 6 to 8 months period was not significant (p=0.26). However, the third model (p=0.004), which included the nine assessments (4 to 8 months), explained 33.33% of the variability of the number of reaches, with 8.75% being attributed to the inclusion of the variable gross motor function.

Three regression models were used to assess the correlation between time of manipulation and gross motor function. In the 4 to 6 months period, there was a significant correlation between the variables (p=0.02, R^2^=0.13), with 3.4% of the variability being attributed to the inclusion of the gross motor function variable in the model. The models that investigated the association in the 6 to 8 months (p=0.4) period and in the total follow-up period (p=0.14) did not show statistical significance.

Figure 3 (A and B) shows the longitudinal progression of the number of reaches, time of manipulation and gross motor function in each child from 4 to 8 months. These data show the uniqueness of the individual developmental profile of hand function and gross motor performance, which are not shown in the results of the inferential analyses because these are based on the behavior of the whole group of children.

**Figure 3A:**
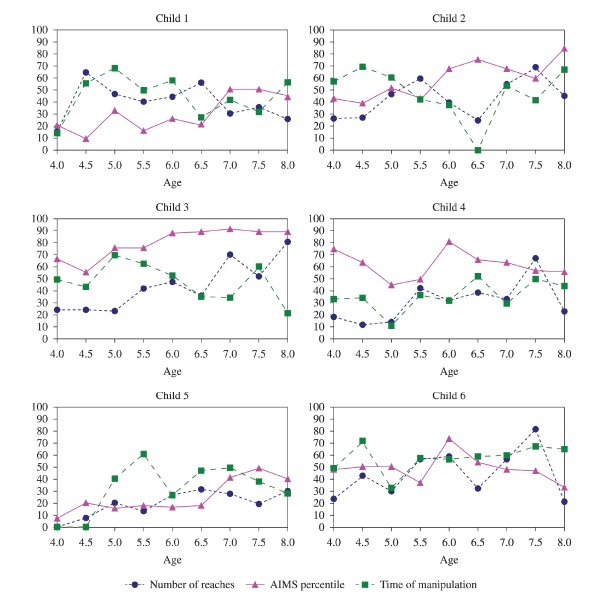
Individual graphic representations of the longitudinal profiles of manual function and gross motor development (AIMS percentile), from each child, from 4 to 8 months.

**Figure 3B:**
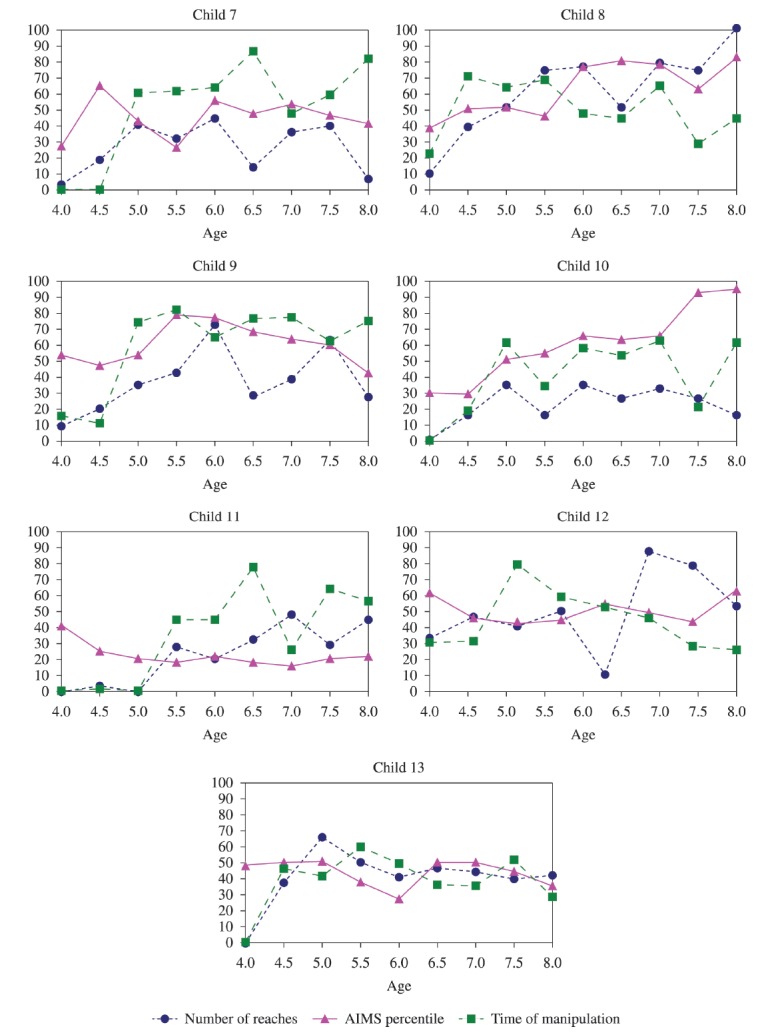
Individual graphic representations of the longitudinal profiles of manual function and gross motor development (AIMS percentile), from each child, from 4 to 8 months.

## Discussion

This study showed that the longitudinal profile of changes in the relationship between hand function and gross motor function of healthy children born at term did not remain constant during the period of 4 to 8 months of age.

### Age effect on hand function

The children reached and manipulated the object concomitantly in the period of 4 to 8 months, and the sequential development of these two hand functions was not shown. Both reaching and manipulation were observed starting at 4 months of age; the reaching and manipulation behaviors seem to consolidate themselves beginning at 5 and 6 months of age, respectively. These ages are similar to those reported by other authors^4^^,^ 7 ^,^ 23.

Some authors have suggested the existence of a sequence in the development of hand functions, such that reaching would precede manipulation of objects^24^^,^ 25. When investigating the development of object manipulation, which is represented by the time the child remained with his or her hand on the object after touching it, our results did not confirm the existence of a sequential order in the development of these two hand functions.

The present study, like others that have assessed reaching development based on the number of reaches performed by the child, documented the continuous increase of this hand function in the first months following its emergence^7^^,^ 22 ^,^ 26. Mathew and Cook^26^ reported a gradual increase in the percentage of efficiency in reaching the object between the ages of 4.5 to 7.5 months when assessing the improvement of the hand trajectory towards the object. These authors concluded that, at the end of 5 months of age, reaching might be considered a consolidated skill in normally developing children^26^.

### Age effect on gross motor function

The results revealed a significant increase in the mean percentile of gross motor development of infants between the 5^th^ and 7^th^ month. This outcome represents the behavior of the group of infants, which was not accurately reflected in the individual development of each child. In fact, the nonlinear profile of changes in gross motor development was clearly observed in the visual analysis of the individual charts that showed the gross motor function and hand function development of each participant (Figures 3A and B). These individual charts described idiosyncrasies in the process of gross motor function and hand function development. When considering the percentile rates of gross motor function, the results show variations in age of peaks, that represent the emergence of skills, as well as variations in ages of valleys, that represent phases of little or no emergence of skills.

These data are consistent with the evidence presented by Darrah et al.^18^ who assessed gross motor development among 45 normally developing children followed monthly from 15 days of life to the beginning of independent gait. The authors observed high variation in the percentile rates of gross motor development of children. Other studies that assessed gross motor development of children in the first year of life found similar results, which supports the argument that changes in the gross motor development of normally developing infants are nonlinear^15^^,^ 27.

### Relationship between hand function and gross motor function

The relationship between the development of hand function and gross motor function was analyzed considering the total follow-up period, as well as at intervals of 4 to 6 and 6 to 8 months. This strategy was adopted because changes were observed in each outcome around the 6^th^ month of age.

In the 4 to 8 month period, there was weak association between hand function (i.e. number of reaches) and gross motor function. However, in the first age interval (i.e. 4 to 6 months), there was significant association between gross motor function and both hand functions, whereas from 6 to 8 months, there was no statistical significance between the outcomes. Therefore, the weak association between the number of reaches and gross motor function, as evidenced in the total follow-up period, became strong only when considering the interval between 4 and 6 months. The lack of association in the 6 to 8 month period may be related to object characteristics that could be explored by reaching as well as by manipulation of the object. In addition, changes in hand functions over time were higher in the 4 to 6 month interval.

Hand function development is related to the child's ability to remain seated without help^10^^,^ 11 ^,^ 28. Thelen and Spencer^29^ investigated the reaching development of four children from 3 weeks to 13 months of age, and the results showed that reaching emerged after the child had developed the ability to keep the head aligned with the torso, as expected for 4-month-old children^29^. These authors related the structural changes of reaching, observed between the 6^th^ and the 7^th^ months, to the child's ability to remain seated without support.

Our results are in agreement with those reported by Darrah et al.^16^, who also observed variation in the correlation levels when assessing the association between gross and fine motor development outcomes of 120 normally developing children aged 9 to 21 months. However, the present study revealed greater variation in the magnitude of association between hand function and gross motor function in the 4 to 8 month interval when compared with the ages assessed by Darrah et al.^16^ Furthermore, while those researchers argued that gross and fine motor skills seem to develop independently, we suggest that the development of these skills may be better characterized as being interdependent.

Considering the relationship between the outcomes assessed in the 4 to 8 month period, some arguments highlighted by Savelsbergh et al.^30^ are supported by the present study. One of them refers to the fact that no single factor should be considered as a priority or determinant for child development. The correlation between these outcomes should be interpreted in a bidirectional manner, i.e., gross motor skills stimulate hand functions whereas the reaching actions and manipulation of an object promote children's gross motor development. This study supports the arguments that the developmental processes are nonlinear by nature and that the variation in development should be considered functional. This finding has relevant implications for both assessment of and intervention in children with delayed motor development^30^.

Studies with longitudinal follow-up of infants present major challenges. The present study did not record relevant experimental losses; only one child did not attend one of the longitudinal assessments. Such characteristics support the internal validity of the results. One limitation of this study was the participation of the examiner who assessed gross motor function in the collection of hand function data; thus, the examiner was not blind for the assessment of both developmental outcomes. However, data extraction of both hand functions from footage was performed after data collection, thus minimizing this bias.

Our results may affect the process of child assessment and identify critical periods for documenting gross motor function and hand function.
